# Sero-prevalence of hepatitis B virus and associated factors among pregnant women in Gambella hospital, South Western Ethiopia: facility based cross-sectional study

**DOI:** 10.1186/s12879-019-4220-z

**Published:** 2019-07-10

**Authors:** Abayneh Tunje Tanga, Misanew Andargie Teshome, Desta Hiko, Chaltu Fikru, Gemechu Kejela Jilo

**Affiliations:** 1 Gambella Reginal Health Bureau, Gambella, Ethiopia; 2 Jimma University institute of health science, Jimma, Ethiopia; 3 Arba Minch University College of health science, Arba Minch, Ethiopia

**Keywords:** Hepatitis B virus, Sero-prevalence, Eugene test strip, Risk factors, Pregnant women, Gambella

## Abstract

**Background:**

Hepatitis B virus (HBV) is a hepatotropic deoxyribonucleic acid (DNA) virus which causes death. More than 300 million people have chronic liver infections globally and about 600,000 people die annually from acute or chronic complications of hepatitis B infection. Recent studies conducted in Ethiopia showed moderate endemicity (3–7.8%) of HBV among pregnant women. However, there is paucity of information on sero- prevalence of HBV and associated factors among pregnant women at Gambella town. The aim of this study is to assess sero-prevalence of hepatitis surface antigen (HBsAg) and associated factors among pregnant women in Gambella Hospital.

**Methods:**

Hospital based cross-sectional study was conducted in a total of 253 pregnant women from March 10–April 15, 2017. Socio-demographic characteristics and risk factors were collected through face to face interview using structured questionnaire. HBV infection was determined using Eugene strip test. Logistic regression analysis was used to determine association between HBsAg sero-positivity and various factors. Findings were presented using 95% CI of Crude Odds Ratios (COR) and Adjusted Odds Ratios (AOR).

**Result:**

The overall sero- prevalence of HBV infection was 7.9% (95% CI, 4.7–11.9), which indicates intermediate endemicity. History of abortion (AOR = 3.56:1: 95% CI, 1.24–10.22), occupation (AOR = 8.36:95% CI, 1.67–41.96) and multiple sexual partner (AOR = 17.38: 95% CI, 4.48–67.49) had statistical significant association with HBsAg sero-positivity.

**Conclusion:**

HBV sero-prevalence in pregnant women shows intermediate endemicity. Hence health education on having single sexual partner and risk factors of abortion should be given. In addition, routine screening and immunization of pregnant women for HBV infection should be strengthen.

## Background

Hepatitis B virus is a hepatotropic deoxyribonucleic acid (DNA) virus which occurs through immune-mediated killing of infected liver cells. It is also recognized as oncogenic virus that can cause a higher risk of developing hepatocellular carcinoma [1]. It is 50–100 times more infectious than HIV [2]. The infection with the hepatitis B virus can be lifelong, causing cirrhosis (liver scarring), liver cancer, liver failure, and death. It can be acute (with discrete onset of symptoms and jaundice or elevated serum ALT > 100 IU/L or chronic with no symptoms. The rate for chronicity is approximately 5% in adult infections, but it reaches 90% in neonatal infections [3, 4].

More than 300 million people have chronic liver infections globally and about 600,000 people die annually from acute or chronic complications of hepatitis B infection. Hepatitis B prevalence is highest in sub-Saharan Africa and East Asia, where between 5 and 10% of the adult population is chronically infected [5, 6].

HBV places a heavy burden on the health care system because of the costs of treatment of liver failure and chronic liver disease (easily reaching up to hundreds of thousands of dollars per person). Chronic viral hepatitis also results in loss of productivity [5].

In countries where HBV is highly endemic (HBsAg prevalence rate of 8% or higher), most infections occur during infancy and early childhood [7]. Recent studies conducted in Ethiopia showed moderate endemicity (3–7.8%) of HBV among pregnant women [8, 9].

Previous studies conducted in different parts of Ethiopia showed that history of use of sharp materials, having multiple sexual partners, ear pricing, abortion, Place of delivery, Genital mutilation, history of tooth extraction cesarean section and tattoo for cosmetics were associated factors for HBs Ag sero-positivity [8, 10–13].

HBV infection during pregnancy is closely related to high risks of maternal complications including: pre-eclampsia, placenta praevia, preterm delivery, placental separation, ante partum haemorrhage, preterm labour, increased incidence of intraventricular haemorrhage, gestational diabetes mellitus and mortality with a high rate of vertical transmission leading to fetal and neonatal hepatitis [14]. Transmission from mother to infant takes place in uterine, during delivery, and after birth. Children born to HBsAg+ and hepatitis e antigen (HBeAg+) mothers have 70–90% chance of prenatal acquisition of HBV infection and over 85–90% of them will eventually become chronic carriers of the disease. Chronic carriers of HBV are main reservoirs for continued transmission of HBV and have a higher risk of hepatocellular carcinoma and liver cirrhosis [1, 15, 16].

Since HBV infected pregnant women are at risk of infecting their babies, knowing magnitude of HBV status and its risk factors in the area is very important. However, there is paucity of information on sero- prevalence of HBV and associated factors among pregnant women at Gambella town. Therefore, the aim of this study is to give deep insight on the magnitude of HBV and its associated factors among pregnant women in anti natal care (ANC) clinic of Gambella hospital.

## Methods

### Study area and period

Hospital based cross sectional study was conducted from March 10–April 15, 2017 in Gambella hospital. Gambella town is the capital of the Gambella regional state located at a distance of 768 k meter in the south west away from Addis Ababa. Gambella Town has a total population of 74.102 of whom 47.2% are women. The town has one hospital, one health center, two governmental junior clinics and 15 private clinics. More than 20 pregnant women visit the hospital ANC clinic per day and get free ANC services.

### Population

The source populations were all pregnant women who visited antenatal care unit at Gambella Hospital during the study period and fulfill selection criteria. All pregnant women whose pregnancy was confirmed by pregnancy test kit are the inclusion criteria and pregnant women who were critically sick and unable to answer questions were excluded from the study.

### Sample size and sampling

The sample size was determined using single population proportion formula with the assumption of 95% confidence interval (CI), Hepatitis B virus prevalence rate 6% [17], degree of precision of 3% and none-response rate of 5%. Finally, the calculated sample size was **253.** The study participants were recruited using non-duplicative consecutive sampling method.

### Data collection

Socio-demographic characteristics and associated risk factors for HBV and HCV infections were collected using structured questionnaire by trained health professionals. The study variables included in this study were maternal age, educational status, occupational status, place of previous birth, residence, abortion, hospital admission, surgical procedure history of blood transfusion, sharp injury, accidental needle stick injuries, splash of body fluids, genital mutilation, human bites, body tattooing, injection-drug use, history of multiple sexual practice and sharing earrings, razors, tooth brushes.

### Detection of HBsAg

Five milliliters of venous blood was collected from each study participant by trained laboratory technologist. Serum was separated by centrifugation at 3000 rpm for 10 min. Each serum was subjected to HBsAg antibody rapid test (Shanghai Eugene Biotech co., Ltd) from Minhang, Shanghai, China, following the manufacturer’s instruction. Eugene rapid test is a qualitative, solid phase, two-site sandwich immunoassay for the detection of HBs Ag in serum HBV infection status **–** was defined by a positive or negative result for HBsAg using HBsAg test strip.

### Data quality assurance

To ensure quality of data, questionnaire was prepared in English language, translated to Amharic and re translated back to English by other person who can speak both languages. To make sure that the questionnaire is appropriate and understandable; it was pre-tested on 5% of pregnant women at Jimma hospital. Training was given for supervisors and data collectors for 1 day. The data collection process was supervised and the collected data were reviewed and checked for completeness by the principal investigator. Then collected data were checked for consistency and accuracy. Standard operating procedures were strictly followed during blood sample collection, storage and analytical process. Storage conditions and expired date of reagents were checked. Positive and negative control sera were run following the manufacturer recommendation of the kit. Finally, reliability was checked by using cronbach alpha (.701).

### Data analysis

Collected data were checked for completeness and consistency, and coded manually. Then data were entered into Ep-idata version 3.1 and cleaned data were exported to SPSS version 21 windows to recode, compute and do other statistical analysis. In the univariate analysis a descriptive statistics was conducted to explore frequency distribution, central tendency, variability (dispersion) and overall distribution of independent variables.

Bivariate logistic regression analysis was conducted to select candidate variables for multivariable analysis. All explanatory variables associated with the outcome variable in bivariate analysis with *p*-value of < 0.25 were included in logistic models of multivariable analysis using backward stepwise method. Adjusted odd ratio along with 95% confidence intervals (CI) was used to check the strength of association. Multicollinearity between the independent variable was checked using variance inflation factor. Finally model fitness was done using Hosmer and Lemeshow Statistics, chi-square (_*x*_2 = 1.82) and *p*-value was 0.61. Variables with *p* value < 0.05 were considered as statistically significant. Confidence interval of the outcome variable was (95%CI; 4.7–11.9).

### Ethical considerations

Ethical approval of the study was obtained from Jimma University institute of health ethical review board. The ethical letter was submitted to Gambella hospital then support letter was obtained from Hospital administration. Written consent was taken after informing the purpose and importance of the study to each participant. To ensure confidentiality of participant’s information, codes were used where by the name of the participant and any identifier of participants was not written on the questionnaire. Participants were interviewed alone to keep the privacy. The participants did not pay for the test. Voluntary Participation was clearly stated that they could choose to participate or not; and they could still receive all the services they usually do if they choose not to participate. Test results were given to the clinicians who were working on ANC clinic and medical and psychological management was given for pregnant women who become positive for the test. The clinical specimen collected during the study period was used only for the stated objectives.

## Result

### Socio-demographic the respondents

Two hundred fifty three (respondent rate of 100%) pregnant women took part in the study. The median age of the study participants was 24 years. One hundred four (41.1%) pregnant women were between the age group of 21 and 25 years. Two hundred thirty nine (94.5%) were urban residence. One hundred forty nine (58.9%) pregnant women were unemployed. One hundred fifty four (60.9%) pregnant women had above secondary education level (≥9) while nineteen (7.5%) were who cannot read and write. Sixty three (30.4%) pregnant women were Oromo while the rest were other ethnic group.

### Sero-prevalence of HBV infection

The overall sero-prevalence of HBV infection was 20 (7.9%), 95% CI; 4.70-11.90). Among these, 7.69% were between 21- 25 years of age. Twelve (15%) of respondents with primary education [1–8], and 7 (4.55%) of respondents with secondary and above (≥9) education had sero-prevalence of HBV infection. Based on their occupation, 18 (12.08%) of pregnant Women who were unemployed and 2 (1.92%) of those who were employed had sero- prevalence of HBV infection (Table 1).From total number of respondents, 26.1% had abortion history and 15(5.9%) had history of multiple sexual partners experience (Table 2).

**Table 1:** Socio-demographic characteristics and sero-prevalence of HBV infection status among pregnant women attending antenatal care at Gambella hospital March 10–April 15, 2017. (*N* = 253)

Variable	Category	Number (%)	HBV		Crude Odds Ratio(95% CI)
			Positive	Negative	
Age	16–20	72 (28.5%)	7 (9.72%)	66 (90.28%)	1.55 (.47–5.13)
	21–25	104 (41.1%)	8 (7.69%)	96 (92.31%)	1.20 (.38–3.82)
	≥26	77 (30.4%)	5 (6.49%)	72 (93.51%)	1
Ethnicity	Oromo	63 (24.9%)	4 (6.35%)	59 (93.65%)	1
	Agnuwak	56 (22.1%)	6 (10.7%)	50 (89.3%)	1.77 (.47,6.63)
	Amhara	43 (17%)	2 (4.65%)	41 (95.35%)	0.72 (.13,4.11)
	Tigre	31 (12.3%)	1 (3.2%)	30 (96.8%)	0.49 (.05,4.59)
	Others	60 (23.7%)	7 (11.7%)	53 (88.3%)	1.95 (.54,7.03)
Educational Status	Cannot read and write	19 (7.5%)	1 (5.26%)	18 (94.74%)	1.18 (.14,10.3)
	1–8	80 (31.6%)	12 (15%)	68 (85%)	3.71 (1.39,9.83)*
	≥9	154 (60.9%)	7 (4.55%)	147 (95.45%)	1
Residence	Urban	239 (94.5)	19 (7.95%)	220 (92.05%)	1
	Rural	14 (5.5)	1 (7.14%)	13 (92.86%)	0.89 (.11,7.18)
Occupation	Unemployed	149 (58.9)	18 (12.08%)	131 (87.92%)	7.01 (1.59,30.89)*
	Employed	104 (41.1)	2 (1.92%)	102 (98.08%)	1

**Table 2:** Sero-prevalence of HBV infection among pregnant women attending antenatal care at Gambella hospital March 10–April 15,2017. (*N* = 253)

Variable	Category	Number (%)	HBV		Crude odds ratio(95% CI)
			Positive	Negative	
Abortion History	Yes	66 (26.1%)	11 (16.67%)	55 (83.33%)	3.96 (1.56,10.04)*
	No	187 (73.9%)	9 (4.8%)	178 (95.2%)	1
Hospital Admission	Yes	61 (24.1%)	4 (6.57%)	57 (93.43%)	0 .77(.25,2.40)
	No	192 (75.9%)	16 (8.33%)	176 (91.67%)	1
History of blood transfusion	Yes	15 (5.9%)	2 (13.33%)	13 (86.67%)	2.24 (.46,10.99)
	No	238 (94.1%)	18 (7.56%)	220 (92.44%)	1
History of surgical procedure	Yes	18 (7.1%)	1 (5.56%)	17 (94.44%)	0 .67 (.08,5.30)
	No	235 (92.9%)	19 (8.08%)	216 (91.92%)	1
Place of previous delivery	No	99 (39.1)	4 (4.04%)	95 (98.08%)	0 .56 (.12,2.62)
	Home	111 (43.9)	13 (11.71%)	98 (88.29%)	1.77 (.48,6.54)
	Health facility	43 (17.0)	3 (6.97%)	40 (93.03%)	1
Multiple sexual partner	Yes	15 (5.9%)	7 (46.67%)	8 (53.33%)	15.14 (4.76,48.24)*
	No	238 (94.1%)	13 (5.46%)	225 (94.54%)	1
Tattoo	Yes	30 (11.9%)	3 (10%)	27 (90%)	1.346 (.37,4.89)
	No	223 (88.1%)	17 (7.62%)	206 (92.38%)	1
Genital mutilation	Yes	153 (60.5)	10 (6.53%)	143 (93.47%)	0.63 (.25,1.57)
	No	100 (39.5)	10 (10%)	90 (90%)	1
Human bite	Yes	47 (18.6)	2 (4.25%)	45 (95.75)	0.46 (.10,2.07)
	No	206 (81.4)	18 (8.73%)	188 (91.27%)	1
Sharp injury	Yes	126 (49.8%)	12 (9.52%)	114 (90.48%)	1.57 (.62,3.97)
	No	127 (50.25)	8 (6.29%)	119 (93.71%)	1
Needle stick injury	Yes	34 (13.4%)	2 (5.88%)	32 (94.12%)	69 (.16,3.15)
	No	219 (86.6%)	18 (8.22%)	201 (91.78%)	1
Sharing of earrings, razors &tooth brush	Yes	11 (4.3%)	2 (18.18%)	9 (81.82%)	2.77 (.56,13.78)*
	No	242 (95.7%)	18 (7.23%)	224 (92.68%)	1
Splash of body fluids	Yes	6 (2.4%)	2 (33.33%)	4 (66.67%)	6.36 (1.09,37.12)*
	No	247 (97.6%)	18 (7.28%)	229 (92.72%)	1

From the variables that were included in the multivariable analysis, abortion, occupation and multiple sexual partners were significantly associated with HBsAg sero-status .However, other variables; educational level, splash of body fluids and sharing of earrings, razors and tooth brush which were candidate in bivariate analysis were not significant. Pregnant women who had abortion history had almost four times higher risk of being sero-positive for HBV infection than those pregnant women who had not abortion history (AOR=3.56:95%CI, 1.24-10.22). Based on multiple sexual partner, pregnant women who had multiple sexual partner were seventeen times more likely to be sero-positive for HBV infection than those pregnant women who had not such partner (AOR=17.38:95%CI, 4.48-67.49). Regarding their occupation, unemployed pregnant women had eight times higher risk of being sero-positive for HBV infection than those who were employed (AOR= 8.36:95CI, 1.67-41.96) (Table 3).

**Table 3:** Factors independently associated with HBV infection among pregnant women attending antenatal care at Gambella hospital from March 10-April 15, 2017. (*N* = 253)

Variable	Category	HBV sero-status		Crude odds	Adjusted odds ratio
		Positive	Negative	ratio(95% CI)	(95% CI)
Abortion	Yes	11 (16.67%)	55 (83.33%)	3.96 (1.56–10.04)	3.56 (1.24,10.22)*
	No	9 (4.8%)	178 (95.2%)	1	1
Multiple sexual Partner	Yes	7 (46.67%)	8 (53.33%)	15.14 (4.756–48.22)	17.38 (4.48,67.49)*
	No	13 (5.46%)	225 (94.54%)	1	1
Occupation	Unemployed	18 (12.08%)	131 (87.92%)	7.01 (1.59–30.89)	8.36 (1.67,41.96)*
	Employed	2 (1.92%)	102 (98.08%)	1	1
Splash of body Fluids	Yes	2 (33.33%)	4 (66.67%)	6.36 (1.09–37.12)	6.79 (1.57,80.25)*
	No	18 (7.28%)	229 (92.72%)	1	1

* = *p*-value< 0.05

## Discussion

The result of this study showed that the prevalence of HBsAg among pregnant women attending antenatal care was 7.9%. According to WHO criteria which classifies endemicity of HBV infection; low endemicity areas (less than 2% sero positive), intermediate endemicity area (2% to 7% sero positive) and high endemicity area (≥8% sero positive), the result showed intermediate endemicity area [18]. The result of this study is in accordance with the hospital based cross-sectional study done at Hawassa University Teaching and Referral Hospital 7.8% and Seroprevalence of hepatitis B surface antigen among pregnant women attending the Hospital for Women & Children in Koutiala, Mali 8% [9, 19]. The result is also almost concordant with the findings found from the study areas of Congo 8.7% [20] and Addis Ababa 6% [17]. Among various risk factors abortion, occupation and multiple sexual partner were statistically significant. However, lower and higher prevalence of HBV infection was assessed in similar study populations in different areas of the world. Areas with high magnitude of HBsAg sero-prevalence include 16.5 % in Nigeria and 10.2% in Cameroon [7, 21]. Some of the areas in which low prevalence of HBV infection detected were 3.1% in Rwanda ,4.3% in Arba Minch and 4.9% in Dessie [9, 12, 22]. Even if the study design is similar, Variations in the prevalence of HBV infection within different parts of the world may be due to differences in the methods used for screening for HBsAg, sample size difference, local government attention for the virus, and cultural and behavioral differences regarding possible risk factors of HBV infection.With regard to socio-demographic status of study participants, high prevalence 7 (9.72%) of HBV infection was observed among pregnant women of age 16 to 20 years and low prevalence observed among those with age of 21 and above years, but the difference was not statistically significant. The observed high prevalence of HBV positivity among younger age group could be defined with the high probability of exposure for high risk health behavior but this is contrasted with other studies because high prevalence of HBV infection was found on the study subjects of age greeter than 20 years [11, 22, 23]. Concerning levels of education, it was noted that high prevalence 12 (15%) of HBV infection was detected among pregnant women who had primary educational [1–8] status and the low prevalence 1 (5.26%) of HBV infection among those who cannot read and write may be due to their low number in the study. Even if it was not statistically significant (*p*-value> 0.05) which made it different from other study reports conducted in Dessie [10], generally the prevalence of HBV infection decreased with increasing levels of education which is in line with other studies conducted in different areas [8, 9]. Although there is no statistical significant difference with residence, pregnant women who were living in urban area had more prevalence19 (7.95%) of HBV infection than those women living in rural which is in line with other study [10, 11]. This may be due to urban women could be engaged in risky life style practices. Based on their occupation, pregnant Women who were unemployed had eight times more likely to be sero-positive for HBV infection than employed. This may be due to that employed pregnant women have good awareness about HBV infection. But this result is in contrast to other results reported from Addis Ababa and Hawassa [8, 9].

According to this study, the prevalence of HBV infection was significantly higher among pregnant women who had history of abortion. The odd of having HBV infection among women with abortion history almost four times higher compared with those who had not abortion history. It is known that, multiple sexual practices may cause unplanned pregnancy which may result abortion and increase the risk of HBV infection if such partners are infected. Accordingly, contaminated instruments used during abortion procedure might increase the probability of acquiring HBV infection. This is similar with a study result reported from Nigeria, Arba Minch, Debre-Tabor and Addis Ababa [7, 12, 17, 24]. When previous place of delivery considered, pregnant women who delivered at home had 13 (11.71%) sero-prevalence of HBV infection while those who delivered at hospital had 3 (6.97%) sero-prevalence. The possible reason may be unsafe delivery practice at home. This is in agreement with a study report from other area of Ethiopia [9]. Except abortion, medical related risk factors like blood transfusion, surgical procedures, place of previous birth and hospital admission were not associated with seropositivity for HBsAg in this study (*p*-value > 0.05). This may be due to the use of standard procedures and disinfected instruments by health professionals. The result is in agreement with previous study conducted at Hawassa, Dessie and Congo [9, 10, 20]. But hospital admission and surgical procedures were statistically significant with high HBsAg sero-prevalence on a studies conducted in Shashemene [25] and Addis Ababa [17] respectively.

Concerning to behavioral and cultural risk factors, pregnant women who had multiple sexual partner had about 17 times higher risk of being sero-positive for HBV infection than pregnant women who had not history of multiple sexual partner. The significant association of having multiple sexual partner with HBV infection was also reported by other investigators [10, 12]. In this study risk factors like tattooing, piercing, genital mutilation and human bites were not associated with seropositivity of HBsAg (*p*-value > 0.05). This is similar with a previous study conducted in Addis Ababa and Hawassa [8, 9],on the other hand, another studies conducted in Debretabor and Nigeria (Osogbo) showed that, in contrast to this study body tattooing was associated with prevalence of HBV infection [7, 13, 24]. This variation may be due to cultural practice differences and different materials used during tattooing.

According to this study, only Sharing of earrings, razors and tooth brush and splash of body fluids were significant during bivariate analysis but in the multivariate analysis none of these accidental risk factors assed were associated with prevalence of HBV infection. Among these risk factors history of sharp injury was statistically significant during a study conducted in Debretabor [24].

Unless preventive measures through vaccination are taken to tackle the risk of transmission, the unborn babies are at a higher risk of contracting HBV infection. The infection was significantly higher among pregnant mothers who had aborted previously and had history of sex with multiple sexual partners. In this study, due to lack of laboratory setup, markers of HBV like HBeAg, HBV-DNA were not detected. Therefore, the major limitation of the study was the new infection of HBV in blood and its active period status not included.

## Conclusion

This study showed that sero-prevalence of HBV infection among pregnant women in Gambella hospital intermediate endemicity. Abortion, occupation and multiple sexual partners significantly associated with HBV infection among pregnant women. Therefore, to halt spread of the virus, health education on modes of transmission like having multiple sexual partners and abortion should be considered. In addition to this, routine screening and immunization of all pregnant women and their infants should be continued in the antenatal and postnatal programs in health facilities.

## Data Availability

The datasets analyzed during the current study are available from the corresponding author on reasonable request.

## Abbreviations

ALT: Alanine Amino Transferase
ANC: Anti Natal Care
CHB: Chronic Hepatitis B
DNA: Deoxyribo Nucleic Acid
HBcAg: Hepatitis Core Antigen
HBeAg: Hepatitis e antigen
HBsAg: Hepatitis B Surface Antigen
HBV: Hepatitis B Virus
HCC: Hepatocellular Carcinoma
HCV: Hepatitis C Virus
HIV: Human Immune Virus
IU/L: International Unit per Litter
SPSS: Statistical Package for Social Sciences
WHO: World Health Organization
